# ﻿Mycobiont-specific primers facilitate the amplification of mitochondrial small subunit ribosomal DNA: a focus on the lichenized fungal genus *Melanelia* (Ascomycota, Parmeliaceae) in Iceland

**DOI:** 10.3897/mycokeys.96.100037

**Published:** 2023-03-21

**Authors:** Maonian Xu, Yingkui Liu, Erik Möller, Scott LaGreca, Patricia Moya, Xinyu Wang, Einar Timdal, Hugo de Boer, Eva Barreno, Lisong Wang, Holger Thüs, Ólafur Andrésson, Kristinn Pétur Magnússon, Elín Soffia Ólafsdóttir, Starri Heiðmarsson

**Affiliations:** 1 Faculty of Pharmaceutical Sciences, University of Iceland, Hofsvallagata 53, IS-107 Reykjavik, Iceland; 2 Jiangsu Key Laboratory of Brain Disease Bioinformation, Research Center for Biochemistry & Molecular Biology, College of Life Sciences, Xuzhou Medical University, 209 Tongshan Road, Xuzhou, 221004, China; 3 Natural History Museum, University of Oslo, NO-0318 Oslo, Norway; 4 Department of Biology, Duke University, NC 27708-0338 Durham, USA; 5 Instituto Cavanilles de Biodiversidad y Biologia Evolutiva (ICBIBE), Dpto. Botánica, Facultat de Ciències Biològiques, Universitat de València, C/ Dr. Moliner 50, 46100-Burjassot, València, Spain; 6 Laboratory for Plant Diversity and Biogeography of East Asia, Kunming Institute of Botany, Chinese Academy of Sciences, 650204 Kunming, China; 7 Botany Department, State Museum of Natural History Stuttgart, D-70191 Stuttgart, Germany; 8 Faculty of Life and Environmental Sciences, University of Iceland, IS-102 Reykjavik, Iceland; 9 Faculty of Natural Resource Sciences, University of Akureyri, IS-600 Akureyri, Iceland; 10 Icelandic Institute of Natural History, IS-600 Akureyri, Iceland; 11 Northwest Iceland Nature Research Centre, IS-550 Sauðárkrókur, Iceland

**Keywords:** *Melanelia*, mtSSU, Parmeliaceae, PCR, primer design

## Abstract

The fungal mitochondrial small subunit (mtSSU) ribosomal DNA is one of the most commonly used loci for phylogenetic analysis of lichen-forming fungi, but their primer specificity to mycobionts has not been evaluated. The current study aimed to design mycobiont-specific mtSSU primers and highlights their utility with an example from the saxicolous lichen-forming fungal genus *Melanelia* Essl. in Iceland. The study found a 12.5% success rate (3 out of 24 specimens with good-quality mycobiont mtSSU sequences) using universal primers (i.e. mrSSU1 and mrSSU3R), not including off-target amplification of environmental fungi, e.g. *Cladophialophoracarrionii* and *Lichenotheliaconvexa*. New mycobiont-specific primers (mt-SSU-581-5’ and mt-SSU-1345-3’) were designed by targeting mycobiont-specific nucleotide sites in comparison with environmental fungal sequences, and assessed for mycobiont primer specificity using *in silico* PCR. The new mycobiont-specific mtSSU primers had a success rate of 91.7% (22 out of 24 specimens with good-quality mycobiont mtSSU sequences) on the studied *Melanelia* specimens. Additional testing confirmed the specificity and yielded amplicons from 79 specimens of other Parmeliaceae mycobiont lineages. This study highlights the effectiveness of designing mycobiont-specific primers for studies on lichen identification, barcoding and phylogenetics.

## ﻿Introduction

In addition to the accepted fungal barcode of nuclear ribosomal internal transcribed spacer (nrITS) locus (Schoch et al. 2012), the fungal mitochondrial small subunit (mtSSU) ribosomal DNA region is one of the most frequently used molecular markers, for two reasons: 1) it has higher mutation rate than its nuclear small subunit counterparts; and 2) it contains both conservative regions that allow for higher taxonomic level analysis, as well as highly variable regions that are suitable for lower taxonomic level analysis. The mtSSU is commonly incorporated into multi-locus phylogenetic analyses of various lichen-forming fungal lineages (Crespo et al. 2007; Nelsen et al. 2011; Divakar et al. 2017; Xu et al. 2020). Due to the utility and popularity of this marker, the paper publishing the universal mtSSU primer pair (i.e. mrSSU1 and mrSSU3R) (Zoller et al. 1999) is well cited (543 times by Feb 15, 2023).

In total, eight universal and conserved regions (i.e. U1 to U8) are recognized in the fungal mtSSU locus (Cummings et al. 1989), and published primer pairs, such as MS1&MS2, NMS1&NMS2, MSU1&MSU7 and mrSSU1&mrSSU3R, were all designed from those universal regions to enable the amplification of a large variety of fungal taxa (Cummings et al. 1989; Zoller et al. 1999; Zhou and Stanosz 2001). They work well for fungal isolates, and even microbial communities like lichens. The most used primer pair in lichen systematics, mrSSU1 and mrSSU3R, is fungus-specific and yields no PCR products from isolated photobionts (Zoller et al. 1999). However, primer specificity to the lichen-forming fungi (mycobionts) has not been evaluated, and the utility of universal mtSSU primers (e.g. mrSSU1 and mrSSU3R) in challenging lichen herbarium specimens, as opposed to freshly collected specimens that are more favorable for PCR, is not well-known. Taking the advantage of the vast number of reference sequences deposited in publicly available databases (e.g. GenBank), *in silico* PCR can be an efficient tool to evaluate primer specificity or potential bias during simulated PCR conditions (Bellemain et al. 2010).

In our recent phylogenetic diversity analyses of Icelandic cetrarioid lichens (Xu et al. 2020), we reported a remarkably low PCR success rate when using the primer pair mrSSU1 and mrSSU3R to amplify herbarium specimens of the saxicolous genus *Melanelia* Essl. (12.5%, 3 out of 24 specimens). For some specimens, instead of the targeted mycobionts, we ended up with good Sanger sequencing results of environmental fungi (unpublished), such as *Cladophialophoracarrionii* (Trejos) de Hoog, Kwon-Chung & McGinnis (Herpotrichiellaceae, Ascomycota) and *Lichenotheliaconvexa* Henssen (Lichenotheliaceae, Ascomycota). This raised questions about primer specificity to the genus *Melanelia*, and mycobionts in general. In the current study, our goal was to design mycobiont-specific mtSSU primers for the genus *Melanelia*, and assess the specificity of these primers to mycobionts. Additionally, we intended to investigate universality of these primers in the Parmeliaceae family using both *in silico* PCR and *in vitro* PCR screening of taxa sampled broadly from specimens across the family.

## ﻿Methods

### ﻿Primer design

Using a multiple sequence alignment, shared primer binding sites were identified in the conserved mtSSU regions among mycobiont genera, that are absent from other ascomycetous fungal genera. Special focus was given to 3’ end unique amplification. The multiple sequence alignment was compiled (Suppl. material 1) from 48 mycobiont mtSSU sequences (10 in-house curated and 38 downloaded from GenBank) and six non-mycobiont/non-lichen-forming fungal sequences of different fungal classes, including *Mycocaliciumsubtile* (Pers.) Szatala (Class: Eurotiomycetes), *Taphrinaflavorubra* W.W. Ray (Class: Taphrinomycetes) and *Botryotiniafuckeliana* (de Bary) Whetzel (Class: Leotiomycetes). Non-mycobiont fungal sequences also include one reference sequence of the ascomycete *Triangulariaanserina* (Rabenh.) X. Wei Wang & Houbraken (Basionym: *Podosporaanserina* (Rabenh.) Niessl; Class: Sordariomycetes; GenBank accession No. X14734), as well as two environmental fungal sequences from *Cladophialophoracarrionii* (Trejos) de Hoog, Kwon-Chung & McGinnis (Class: Eurotiomycetes) and *Lichenotheliaconvexa* Henssen (Class: Dothideomycetes), both of which were found to co-inhibit with *Melanelia* mycobionts. Melting temperature and primer dimer formation were estimated using Multiple Primer Analyzer (ThermoFisher, MA, USA).

### ﻿*In silico* PCR

EcoPCR (Ficetola et al. 2010) was used for simulated *in silico* amplification of the mtSSU locus and also to verify amplicon possibilities against an in-house reference ecoPCR database containing overall 2,233,856 fungal sequences. We followed the published procedure (Bellemain et al. 2010) to construct the in-house reference database: all fungal sequences were downloaded from the EMBL fungal database of standard targeted annotated assembled sequences (STD), and sequences were annotated using NCBI taxonomy. Data containing the vast number of annotated fungal sequences were transformed into ecoPCR format before *in silico* simulation. Two pairs of primers were tested: the commonly used pair mrSSU1 and mrSSU3R, and our newly designed mycobiont-specific primer pair mt-SSU-581-5’ and mt-SSU-1345-3’. In the setting of simulations, amplicon sizes were accepted between 200 bp to 2500 bp, and only up to three nucleotide mismatches between primers and templates were allowed (except for the last two positions at the 3’ end), according to described parameters (Bellemain et al. 2010; Riaz et al. 2011; Liu and Erséus 2017).

### ﻿Taxon sampling and DNA extraction

The current study included 24 *Melanelia* herbarium specimens collected from 1997 to 2014, consisting of *M.agnata* (n=8), *M.hepatizon* (n=12) and *M.stygia* (n=4), all of which were morphologically identified and verified with fungal nrITS DNA barcoding and chemotaxonomic analyses in a previous study (Xu et al. 2017). In addition to the *Melanelia* specimens, 79 specimens of other genera in the same family were also included to test primer universality in the family. The specimen list is provided in Appendix 1. Visible substrates attached to thalli were removed with sterile tweezers or brushes before DNA extraction. Whole genomic DNA was extracted from lichen thalli (ca. 15–20 mg per specimen) using the CTAB method (Cubero et al. 1999).

### ﻿*In vitro* PCR and sequence analysis

The PCR master mix and thermal cycler conditions were followed from our published protocol (Xu et al. 2020). Two touchdown programmes were used, where the annealing temperature ramp 61–57 °C (decreasing 1 °C per cycle) was used for mrSSU1 and mrSSU3R, and 54–50 °C for the newly designed primer pair, mt-SSU-581-5’ and mt-SSU-1345-3’, according to predicted melting temperatures (Table 1). Presence and sizes of amplicons were determined by performing 2% agarose gel electrophoresis, using SYBR safe stain (Invitrogen, CA, USA). Amplicons showing single bands were purified with ExoSAP (Fermentas Inc., Hanover, MD, USA) and sequenced in both directions using Sanger sequencing (Macrogen Europe BV, the Netherlands). The same primers were used for both PCRs and Sanger sequencing.

**Table 1.** Primers used for the amplification of the mtSSU locus.

Primer^a^	Location^b^	Sequence 5’ – 3’	T_m_^c^	Reference
**Major primers**				
mrSSU1(F)	533-552	AGCAGTGAGGAATATTGGTC	58.7	Zoller et al. 1999
mt-SSU-581-5’(F)	581-600	GGAGGAATGTATAGCAATAG	53.5	This study
mt-SSU-862-5’(F)^d^	862-880	GAAAGCATCYCCTTATGTG	56.7	This study
mt-SSU-1345-3’(R)	1345-1324	CGCTTGTAAATATATCTTATTG	53.4	This study
mrSSU3R(R)	1524-1505	ATGTGGCACGTCTATAGCCC	64.2	Zoller et al. 1999
**Alternative primers**				
mt-SSU-574-5’(F)	574-594	GCAACTTGRARGAATGTATAG	56.0	This study
mt-SSU-897-3’(R)	897-880	CCCTCAACGTCAGTTATC	56.0	This study
mt-SSU-1093-3’(R)	1093-1073	TCTAATGATTTCARTTCCAA	55.3	This study
mt-SSU-1372-3’(R)	1372-1353	CGACATTAACTGAAGACAGC	58.1	This study
mt-SSU-1492-3’(R)	1492-1472	CCATGATGACTTGTCTTAGTC	56.8	This study
mt-SSU-1548-3’(R)	1548-1529	ATTTCACACCCTTTTGTAAG	56.3	This study

^a^: primer nomenclature follows the recommendation (Gargas and DePriest 1996). Forward or reverse primers are indicated by (F) or (R); ^b^: location is relative to the reference fungal mtSSU sequence with GenBank accession No. X14734; ^c^: melting temperature (T_m_) is estimated using the multiple primer analyzer online tool; ^d^: the primer mt-SSU-862-5’ is recommended for herbarium specimens, focusing on the amplification of the highly variable region between U5 and U6 (numbers of variable sites refer to Table 3).

Ambiguous sequences at both ends of the raw sequencing data were trimmed with the software PhyDE v0.9971. Sequence contigs were assembled from both directions and ambiguous base calling was checked. Sequences were identified by BLAST searches. Successful PCR amplification was defined as on-target/mycobiont-specific amplification and clean mycobiont mtSSU sequences without ambiguous base calling. Success rates in percentages were calculated as the number of specimens with successful PCR amplification divided by the total number of specimens. Multiple sequence alignment was performed using MAFFT (Katoh and Standley 2013) and then manually adjusted.

## ﻿Results

### ﻿Primer design

**Figure 1.**
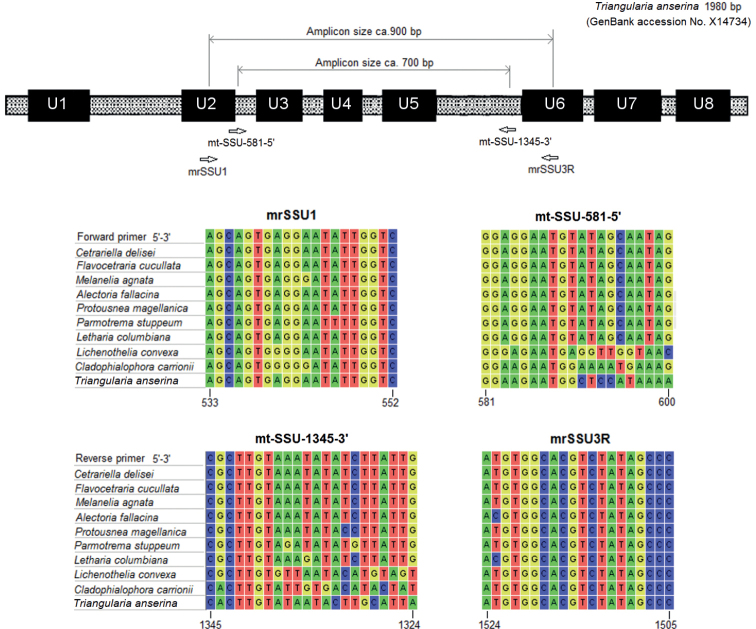
Primer design and sequence alignments at the priming locations. Conservative regions (i.e. U1 to U8) are marked as previously designated (Cummings et al. 1989) with adjustments from the reference. Nucleotide sites are relative to the 1980 bp-long sequence of the fungus *Triangulariaanserina* (GenBank accession No. X14734).

Multiple sequence alignments at the primer binding sites are shown in Fig. 1. The universal primers mrSSU1 and mrSSU3R were designed at the conserved region U2 and U6, respectively, with an expected amplicon size of around 900 base pairs (bp). From the alignments, this primer pair shows little discriminating power between lichen-forming and environmental ascomycetes. Therefore, searching for mycobiont-specific priming sites in the universal regions was not possible, and more variable regions were checked. New primers were designed at the genetic regions where high discriminations were found, particularly at the 3’ end. The new forward primer is located at the variable sites between U2 and U3, showing ca. nine nucleotide differences between Parmeliaceae and environmental fungi. Similarly, the reverse primer was designed at the connecting zone between U5 and U6 with potential discriminating power including roughly nine nucleotide differences.

The newly designed primers, mt-SSU-581-5’ with mt-SSU-1345-3’, were named according to the primer nomenclature recommendation (Gargas and DePriest 1996): “mt-SSU” indicates the mitochondrial small subunit ribosomal DNA, and -5’ or -3’ defines the primer annealing to the coding strand (-5’ for forward primers) or the non-coding one (-3’ for reverse primers). The number before -5’ or -3’ is the nucleotide position relative to the reference sequence of the fungus *Triangulariaanserina* at the 5’ end, so these numbers help the estimation of amplicon sizes. For instance, mt-SSU-581-5’ with mt-SSU-1345-3’ will result in amplicons estimated around 700 bp. The numbering of primers, i.e. 581 and 1345, is based on the reference fungus *Triangulariaanserina*, which gives rise to an amplicon size of 764 bp. However, amplicons of ParmeliaceaemtSSU are usually shorter the reference fungus, as seen from Suppl. material 1.

### ﻿*In silico* primer specificity

**Figure 2.**
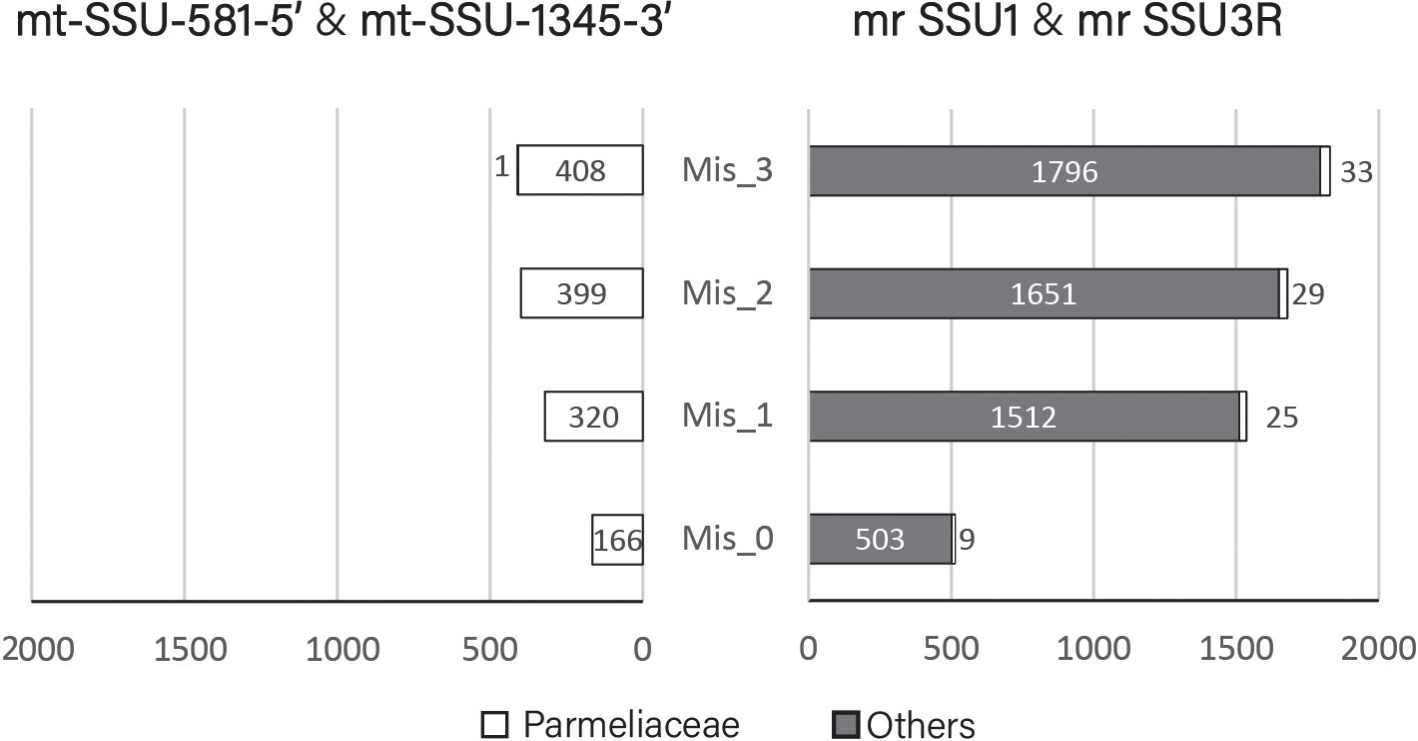
Comparison of primer specificity between the new and the universal primer pairs using *in silico* PCR. The number of off-target amplifications is classified as others in grey, while desired amplifications of lichen-forming fungi in Parmeliaceae are marked in white. The number of nucleotide mismatches in the priming sites is shown as Mis_0 to Mis_3, indicating 0 to 3 nucleotide mismatches.

The amplification success was significantly affected by the allowed number of mismatches and positions between archived fungal sequences and primers (Fig. 2). As more mismatches between primers and templates are allowed, the numbers of amplicons increase. The amplicon profiles between the new and universal primer pairs are considerably different. Universal primers give rise to an overwhelming proportion (ca. 98%) of sequences from non-Parmeliaceae fungi, regardless of the number of nucleotide mismatches. Our *in silico* results show that the amplicons are mainly from three fungal families in the example of three mismatches: Nectriaceae (n=589), Aspergillaceae (n=201) and Trichocomaceae (n=156). A full list of in silico amplicons is provided in Suppl. material 2. In contrast, the new primer pair only produced sequences of Parmeliaceae fungi when less than two mismatches were allowed, and only a single non-Parmeliaceae fungal sequence was amplified with three mismatches.

### ﻿PCR screening and *in vitro* validation

**Figure 3.**
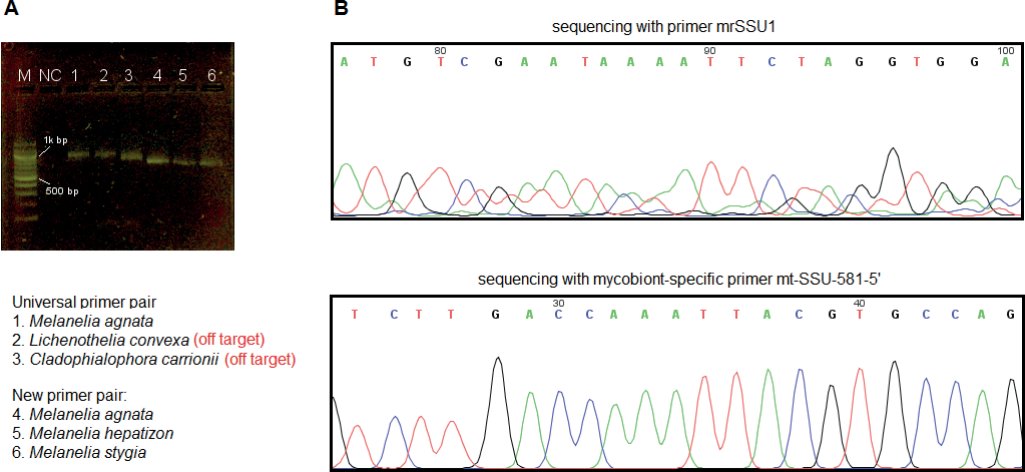
PCR amplification and sequencing results of the mtSSU locus in the genus *Melanelia***A** 2% agarose gel electrophoresis of PCR products, where lanes 1-3 contain amplicons from the universal primer pair mrSSU1 and mrSSU3R, and lanes 4-6 are from the mycobiont-specific primer pair mt-SSU-598-5’and mt-SSU-1324-3’. Abbreviations: M-molecular ladder 100 bp; NC-negative control **B** illustration of sequencing results (*M.agnata*, voucher number LA29683) using the forward primers: the universal primer mrSSU1 (upper) and the mycobiont-specific primer mt-SSU-581-5’ (lower), respectively.

Amplicons resulting from the universal primer pair mrSSU1 and mrSSU3R are around 1000 bp in length (lanes 1–3 in Fig. 3A). However, after sequencing, only the amplicons in lane 1 are identified as the lichen-forming fungus *M.agnata* after BLAST search, while lanes 2 and 3 are off-target amplification of non-lichen-forming fungi, *Lichenotheliaconvexa* (GenBank accession No. OQ450499) and *Cladophialophoracarrionii* (GenBank accession No. OQ450500), respectively. The amplicon of *C.carrionii* is slightly shorter than the other two. Among 24 *Melanelia* specimens, we only obtained three sequences from the mycobionts (3/24, 12.5% success), while the others showed messy and ambiguous base calling or even no PCR products in gel electrophoresis. Fig. 3B shows Sanger electropherograms resulting from the same DNA extract of one *M.agnata* specimen but different primers during PCR and sequencing, and the importance of mycobiont-specific primer is highlighted in generating good-quality sequences. Amplicons from the newly designed primers were around 700 bp long. The resulting Sanger electropherograms show unambiguous nucleotides with good quality (Fig. 3B, lower electropherogram), and we obtained 22 mycobiont sequences out of 24 specimens (22/24, 91.7% success). The remaining two specimens yielded no bands after PCR.

Using the new primers, the mtSSU region was also successfully amplified from DNA extracts of other genera in Parmeliaceae (Table 2), e.g. *Alectoria*, *Evernia*, *Flavoparmelia*, *Xanthoparmelia*, etc. The exception is the genus *Usnea*, from which we did not succeed with the newly designed primers (mt-SSU-581-5’ and mt-SSU-1345-3’) or universal primers (mrSSU1 and mrSSU3R). To solve the amplification problem in *Usnea*, a multiple sequence alignment (Suppl. material 3) was specially made for this genus, which contained 13 reference sequences: six partial mtSSU sequences from PCR, and seven mitochodrial genomes containing the whole mtSSU region. The alignment in (Suppl. material 3) shows that: 1) there are unique and long introns inserted in U4 (e.g. 907 bp for *U.trachycarpa* (Stirt.) Müll.Arg. and *U.antarctica* Du Rietz), U5 (e.g. 757-1110 bp for *U.halei* P.Clerc, *U.subgracilis* Vain. and *U.ceratina* Ach.) and U6 (e.g. 535–848 bp for *U.subfusca* Stirt., *U.subscabrosa* Nyl. ex Motyka, *U.ceratina*, *U.pennsylvanica* Motyka and *U.subgracilis*) regions, and thus when the primers mrSSU1 and mrSSU3R are used, the estimated size of PCR amplicons may reach ca. 3000 bp; 2) there is a high nucleotide variation in the primer binding sites for the newly designed mycobiont-specific primers mt-SSU-581-5’ and mt-SSU-1345-3’, which prohibit primer binding, especially at the 3’ end (Suppl. material 2: fig. S1).

**Table 2.** PCR amplification summary in different lichen groups using newly designed primers.

Lichens	Number of amplified/sampled specimens
**Cetrarioid**	
*Cetraria* clade^a^	18/18
*Nephromopsis* clade^a^	6/6
*Melanelia*	22/24
**Parmelioid**	
*Flavoparmelia*	5/5
*Melanelixia*	4/5
*Melanohalea*	9/11
*Parmotrema*	2/2
*Xanthoparmelia*	4/4
**Others**	
*Alectoria*	9/9
*Evernia*	11/11
*Protousnea*	1/1
*Usnea*	6/7^b^

^a^: *Cetraria* and *Nephromopsis* clades follow the circumscription of Divakar et al. (2017); ^b^: Amplification for the *Usnea* genus used alternative primers in Table 1.

Therefore, we designed alternative primers (the pair mt-SSU-574-5’ and mt-SSU-897-3’) for the amplification of shorter mtSSU sequences (ca. 400 bp) in *Usnea*, to avoid amplifying the introns in U4 or U5 region. For *Usnea* species lacking the intron in the U4 region, we recommend the the primer pair mt-SSU-574-5’ and mt-SSU-1093-3’, which produces amplicons as long as ca. 500 bp. These primers are also useful for old herbarium specimens, for which longer amplicons can not be obtained. The same PCR condition was used for the genus *Usnea*, except for the adjustment of annealing temperatures: 56–52 °C for touchdown cycles (decrease 1 °C per cycle), and 52 °C for the last 30 to 32 cycles. The primers (mt-SSU-574-5’ and mt-SSU-897-3’/mt-SSU-1093-3’) have been tested with *in vitro* PCR screening and we got six mycobiont mtSSU sequences out of seven *Usnea* specimens. The improved success rate is at the cost of variable sites after the U5 zone. The reverse primers, mt-SSU-897-3’ or mt-SSU-1093-3’, can also be used with the mycobiont-specific forward primer mt-SSU-581-5’ for other genera in Parmeliaceae.

We also provide two universal reverse primers (i.e. mt-SSU-1372-3’ and mt-SSU-1492-3’) to replace mt-SSU-1345-3’ when the latter is not working. In this case, we recommend combining a mycobiont-specific forward primer, either mt-SSU-574-5’ or mt-SSU-581-5’, to enhance the specificity for PCR. Two reverse primer mt-SSU-1492-3’ and mt-SSU-1548-3’ were designed to replace the published primer mrSSU3R, for two reasons: 1) the primer mrSSU3R has a much higher melting temperature (T_m_ 64.2 °C) than the newly designed primers, which have their T_m_ around 55 °C; 2) mrSSU3R has four consecutive G/C at the 3’ end with a higher risk of non-specific binding.

Notably, the region between U5 and U6 has the highest number of variable sites (Table 3). For the genus *Melanelia*, nearly all variable sites (18 out of 22 variable nucleotide sites) come from this region, which is the most informative for specimen discrimination. Therefore, we designed an alternative forward primer, mt-SSU-862-5’ in combined use with the reverse primer mt-SSU-1345-3’, which focuses on the amplification of the region between U5 and U6. Although a fairly short amplicon size around 450 bp is obtained from this primer pair (mt-SSU-862-5’ and mt-SSU-1345-3’), it actually contains the majority of the total variable sites (Table 3), which is the most informative region of the mtSSU locus. We have obtained successful PCR products and good sequencing results (i.e. clean mycobiont mtSSU sequence without ambiguous base calling) with this primer pair from specimens which failed in obtaining longer amplicons.

**Table 3.** Numbers of variable sites between mtSSU universal regions in selected genera. Numbers are shown as variable nucleotide sites/total nucleotide sites.

Lichens	Number of variable sites (bp)			
	U2-U3^a^	U3-U4^a^	U4-U5^a^	U5-U6^a^
**Cetrarioid**				
*Cetraria* clade^b^	11/107	17/166	1/39	26/247
*Nephromopsis* clade^b^	6/107	9/166	4/39	19/249
*Melanelia*	1/107	3/166	0/39	18/233
**Others**				
*Alectoria*	2/107	14/167	1/39	37/258
*Evernia*	4/107	9/171	0/39	21/224
*Flavoparmelia*	4/107	34/166	1/39	45/241

^a^: Universal regions (U2-U6) refer to designations in Fig. 1; ^b^: *Cetraria* and *Nephromopsis* clades follow the circumscription of Divakar et al. (2017).

## ﻿Discussion

Of all PCR optimization approaches, primer design is a critical but usually neglected factor, since one tends to pick up the primers from existing literature (Ekman 1999). Universal primers may show good performance in some lichen taxa, presumably with freshly collected specimens. However, with regards to Icelandic saxicolous *Melanelia* lichens, we demonstrated low success rate using universal primers (Xu et al. 2020), since off-target amplification is prone to happen in microbial communities like lichens. Primer design should be incorporated as an essential part of Sanger sequencing-based molecular systematics studies. This is facilitated by the deposition of large amounts of sequence data in publicly available databases that can be used for designing mycobiont-specific primers.

Selecting primer binding sites in variable (e.g. the region between U2 and U3 for forward primer, the region between U5 and U6 for reverse primer) instead of conserved regions (e.g. U2, U6) will favour the design of mycobiont-specific primers, while still keeping most variable sites, in comparison with the often used primer pair mrSSU1 (designed at U2) and mrSSU3R (designed at U6). In the latter primer pair, the universal regions (i.e. U2 and U6) are highly conserved, and few nucleotide variations are present at the species level. Amplification of universal sites at U2 and U6 may help sequence alignment, but it will not add a significant number of variable sites. Instead, targeting a shorter amplicon with enhanced primer binding to mycobiont DNA templates will conceivably increase the PCR success, particularly for herbarium specimens which contain degraded DNA templates (Kistenich et al. 2019).

Regions between universal sites (e.g. the region between U5 and U6 in Fig. 1) have been shown to have high variations in sequence lengths (Zoller et al. 1999). These noticeable differences in sequence length could explain the observed off-target PCR amplification with universal primers, in which the fungal templates with shorter amplicons are preferentially targeted. Our alignment shows that the amplified environmental fungi have shorter mtSSU amplicons (by ca. 50–100 bp) than the lichen-forming fungi, and the largest sequence length differences reside in the region between U5 and U6 (see Suppl. material 1).

Our *in vitro* PCR tests only compared the effectiveness of new primers with the most commonly used primer pair – mrSSU1 and mrSSU3R, instead of other known mtSSU primers, such as MS1&MS2 (White et al. 1990), NMS1&NMS2 (Li et al. 1994) and MSU1&MSU7 (Zhou and Stanosz 2001). The reason why we did not include MS1&MS2, NMS1&NMS2 are twofold: 1) these primers were designed as universal primers for different fungal lineages (see alignment in Suppl. material 1), not specifically designed for lichenized ascomycetes (Suppl. material 2: fig. S2), and 2) these two primer pairs were designed to amplify the conserved region between U2 and U5, neglecting the most informative region between U5 and U6 for specimen identification (Table 3). The reason why we excluded MSU1&MSU7 is also twofold: 1) they are not mycobiont-specific (Suppl. material 2: fig. S2), and 2) they will lead to impractically long amplicons for Sanger sequencing, which are close to 2000 bp for most Parmeliaceae and over 3000 bp for intron-rich *Usnea* species.

Co-amplification of non-lichen-forming fungi revealed the intrinsic complexity and habitat ecology of lichen symbiosis (Banchi et al. 2018; Gueidan et al. 2019; Smith et al. 2020). For instance, the amplified fungus *Lichenotheliaconvexa* is a known saxicolous and lichenicolous fungus, often co-inhabiting with lichen-forming fungi (Kocourková and Knudsen 2011). The other amplified fungus, *Cladophialophoracarrionii*, is mostly found on decaying plants, but it has also been reported in association with lichens (Diederich et al. 2013).

In addition to our success in the genus *Melanelia*, the new primers also gave good results on other genera in the family Parmeliaceae, indicating good primer universality in Parmeliaceae. The only exception is the genus *Usnea*, which is intron-rich and more variable at primer binding sites. This explains why the mtSSU locus was not included in recent phylogenetic studies of the genus *Usnea* (Mark et al. 2016; Gerlach et al. 2019; Ohmura 2020). To this end, we designed alternative mtSSU primers (Table 1), to target shorter amplicons and to avoid amplification of introns (Suppl. material 3). For unsampled Parmeliaceae taxa, we expect that the mycobiont-specific primers as well as alternative primers will also work out.

The Parmeliaceae sequences amplified with the universal primer pair may be underestimated in the simulation of *in silico* PCR. Theoretically, the shorter amplicons using newly designed primers are more likely to be amplified than longer amplicons with the universal primers. Some submitted mtSSU sequences may contain neither the forward nor reverse primer binding sites, and thus are not sufficiently long to be served as *in silico* PCR templates. Therefore, amplification would fail with the universal primers using *in silico* PCR for these samples. Relying on the number and position of primer-template mismatches alone may be insufficient for *in silico* PCR; however, the *in silico* results coincide with the *in vitro* PCR results. Here, we have validated the higher specificity of the newly designed primers compared to universal primers during *in vitro* PCR. Therefore, *in silico* specificity check of primers followed by *in vitro* analysis is recommended to confirm the appropriate choice of primers, thus preventing the amplification of unspecific sequences and ensuring appropriate amplification of target sequences.

Our mtSSU sequence data can be incorporated into multi-locus phylogenetic analyses to assess species relationship in the genus *Melanelia*, for which a phylogeny has yet to be reconstructed. Using the nuclear ribosomal internal transcribed spacer (nrITS) marker, previous fungal barcoding studies have detected multiple haplotypes within *Melanelia* species, and hypervariability of the nrITS regions suggest the presence of hidden species diversity (Leavitt et al. 2014; Xu et al. 2017; Szczepańska et al. 2021), which must be tested using multi-locus phylogenetic analyses. Before PCR amplification of additional mycobiont loci, however, precautions must be taken to make sure that the mycobiont DNA templates are targeted, as we have shown in the current study. It is expected that more mycobiont-specific primers will be designed for other loci (e.g. RPB2 and MCM7), after which species relationship can be assessed by reconstructing multi-locus phylogenies. Chemotaxonomic tools can also be applied to aid in species delimitation (Xu et al. 2016, 2017).

## ﻿Conclusion

Here we demonstrate an efficient and effective approach for successful PCR amplification. We designed mycobiont-specific mtSSU primers, which significantly enhanced the successful PCR rate from 12.5% to 91.7% for Icelandic *Melanelia* lichens. Moreover, the primers show strong specificity within the family Parmeliaceae. This study emphasizes the importance of thoughtful primer design in molecular systematics studies of lichen-forming fungi.

## ﻿Appendix 1

**Table A1.** Voucher information and GenBank accession numbers of the amplified mtSSU loci by newly designed primers. ^a^: *Usnea* specimens were amplified with alternative primers.

Species	Location	Collection date	Herbarium number	GenBank accession number
*Alectoriamexicana*	Mexico: Jalisco	2009-Jan-14	0197880 (DUKE)	OP901526
*Alectoriaochroleuca*	Iceland: INo	2012-Jul-26	LA32005 (AMNH)	OP901527
*Alectoriaochroleuca*	Iceland: IVe	2020-Oct-9	LA32013 (AMNH)	OP901528
*Alectoriaochroleuca*	Iceland: INo	1997-May-18	LA28088 (AMNH)	OP901529
*Alectoriaochroleuca*	China: Yunnan	2012-Sep-10	L35822 (KUN)	OP901530
*Alectoriasarmentosa*	Norway: Trondelag	2018-Aug-6	LF00037 (AMNH)	OP901531
*Alectoriasarmentosa*	Iceland: IVe	2013-Jul-23	LA32003 (AMNH)	OP901532
*Alectoriasarmentosa*	Iceland: INo	2012-Aug-21	LA32002 (AMNH)	OP901533
*Alectoriasarmentosa*	Iceland: INo	2006-Jul-3	LA30049 (AMNH)	OP901534
*Allocetrariaflavonigrescens*	China: Yunnan	2015-Nov-1	L52601 (KUN)	OP901604
*Cetrariellafastigiata*	Norway: Malselv	2011-Sep-12	L177156 (O)	OP901535
*Cetrariellafastigiata*	Norway: Finnmark	2014-Jul-4	L195985 (O)	OP901536
*Cetrariellafastigiata*	Norway: Finnmark	2011-Jun-23	L170481 (O)	OP901537
*Cetrariellafastigiata*	Norway: Hedmark	2018-Aug-16	L208163 (O)	OP901538
*Cetrariaericetorum*	Iceland: INo	2016-Aug-29	LA31901 (AMNH)	OP901539
*Cetrariaericetorum*	Iceland: INo	2010-Sep-10	LA31538 (AMNH)	OP901540
*Cetrariaislandica*	Poland: Jelenia Gora	2017-Aug-26	SMNS-STU-F 0005174 (STU)	OP901541
*Cetrariaislandica*	Germany: Feldberg	2017-Aug-15	SMNS-STU-F 0000549 (STU)	OP901542
*Everniadivaricata*	USA: Utah	2006-Aug-10	0188304 (DUKE)	OP901543
*Everniadivaricata*	Austria: Salzburg	2019-Sep-2	SMNS-STU-F 0004925 (STU)	OP901544
*Everniamesomorpha*	Norway: Innlandet	2009-Sep-17	L158139 (O)	OP901545
*Everniamesomorpha*	Norway: Viken	2014-Oct-25	L200008 (O)	OP901546
*Everniamesomorpha*	Canada: Ontario	2015-July-13	0405706 (DUKE)	OP901547
*Everniamesomorpha*	China: Yunnan	2018-Sep-27	L64081 (KUN)	OP901548
*Everniamesomorpha*	China: Inner Mongolia	2011-Jun-1	L24002 (KUN)	OP901549
*Everniamesomorpha*	China: Yunnan	2017-Jul-8	L58746 (KUN)	OP901550
*Everniaprunastri*	Norway: Hordaland	2011-Jul-27	L194342 (O)	OP901551
*Everniaprunastri*	USA: Idaho	2009-Oct-4	0154766 (DUKE)	OP901552
*Everniaprunastri*	Spain: Castellon	2007	LF00002 (AMNH)	OP901553
*Flavocetrariacucullata*	Iceland: INo	2002-Jul-29	LA28953 (AMNH)	OP901554
*Flavocetrariacucullata*	Iceland: INo	2000-Aug-1	LA28174 (AMNH)	OP901555
*Flavocetrariacucullata*	Norway: Buskerud	2015-Jun-16	L200903 (O)	OP901556
*Flavocetrariacucullata*	Norway: Buskerud	2013-Sep-29	L184721 (O)	OP901557
*Flavoparmeliacaperata*	Spain: Galicia	-	LF00008 (AMNH)	OP901558
*Flavoparmeliacaperata*	Spain: Vigo	-	LF00013 (AMNH)	OP901559
*Flavoparmeliasoredians*	Spain: Pontevedra	2015-Aug-10	LF00004 (AMNH)	OP901560
*Flavoparmeliasoredians*	Spain: Pontevedra	2015-Aug-10	LF00005 (AMNH)	OP901561
*Flavoparmeliasoredians*	Spain: Castellon	2017	LF00007 (AMNH)	OP901562
*Melaneliaagnata*	Iceland: IMi	1999	LA29195 (AMNH)	OP901563
*Melaneliaagnata*	Iceland: IMi	2002-Aug-7	LA29683 (AMNH)	OP901564
*Melaneliaagnata*	Iceland: INo	2005-Jun-28	LA27562 (AMNH)	OP901565
*Melaneliaagnata*	Iceland: IAu	2008-Oct-1	LA30974 (AMNH)	OP901566
*Melaneliaagnata*	Iceland: INo	2012-Jun-27	LA31859 (AMNH)	OP901567
*Melaneliaagnata*	Iceland: IMi	1999-Aug-11	LA27454 (AMNH)	OP901568
*Melaneliaagnata*	Iceland: IMi	2000-Aug-11	LA26648 (AMNH)	OP901569
*Melaneliaagnata*	Iceland: IMi	1998-Aug-1	LA33428 (AMNH)	OP901570
*Melaneliahepatizon*	Iceland: IAu	2003-Jul-24	LA30501 (AMNH)	OP901571
*Melaneliahepatizon*	Iceland: IAu	1997-Jul-19	LA27296 (AMNH)	OP901572
*Melaneliahepatizon*	Iceland: IVe	2007-Aug-23	LA30676 (AMNH)	OP901573
*Melaneliahepatizon*	Iceland: INv	2007-Aug-24	LA30674 (AMNH)	OP901574
*Melaneliahepatizon*	Iceland: IVe	2007-Aug-23	LA30675 (AMNH)	OP901575
*Melaneliahepatizon*	Iceland: INv	2007-Aug-24	LA30673 (AMNH)	OP901576
*Melaneliahepatizon*	Iceland: INo	2014-Jun-26	LA20781 (AMNH)	OP901577
*Melaneliahepatizon*	Iceland: IAu	1998-Aug-25	LA30117 (AMNH)	OP901578
*Melaneliahepatizon*	Iceland: INv	2012-Jul-25	LA31861 (AMNH)	OP901579
*Melaneliahepatizon*	Iceland: INo	2012-Jun-25	LF00036 (AMNH)	OP901580
*Melaneliastygia*	Iceland: IAu	1998-Aug-25	LA19972 (AMNH)	OP901581
*Melaneliastygia*	Iceland: IAu	2000-Jul-20	LA28243 (AMNH)	OP901582
*Melaneliastygia*	Iceland: IAu	2014-Jun-10	LA20775 (AMNH)	OP901583
*Melaneliastygia*	Iceland: IAu	2013-Jul-19	LA16894 (AMNH)	OP901584
*Melanelixiafuliginosa*	Iceland: IVe	2005-Jul-21	LA27514 (AMNH)	OP901585
*Melanelixiafuliginosa*	Iceland: INv	2005-Jul-6	LA27518 (AMNH)	OP901586
*Melanelixiafuliginosa*	Iceland: INv	2013-Jul-8	LA16895 (AMNH)	OP901587
*Melanelixiafuliginosa*	Iceland: INo	2014-Jun-25	LA20777 (AMNH)	OP901588
*Melanelixiasubaurifera*	Iceland: IAu	2001-May-26	LA27950 (AMNH)	OP901597
*Melanohaleaexasperata*	Iceland: IAu	1997-Aug-6	LA27384 (AMNH)	OP901589
*Melanohaleaexasperata*	Iceland: IAu	2001-May-25	LA27958 (AMNH)	OP901590
*Melanohaleaexasperatula*	Iceland: INo	2012-Sep-5	LA31766 (AMNH)	OP901591
*Melanohaleainfumata*	Iceland: INo	2007-Jun-8	LA30618 (AMNH)	OP901592
*Melanohaleainfumata*	Iceland: INo	2007-Apr-29	LA30623 (AMNH)	OP901593
*Melanohaleaolivacea*	Iceland: INo	2010-Jun-29	LA31446 (AMNH)	OP901594
*Melanohaleaseptentrionalis*	Iceland: IAu	1997-Aug-6	LA27382 (AMNH)	OP901595
*Melanohaleaseptentrionalis*	Iceland: IAu	2001-May-25	LA27954 (AMNH)	OP901596
*Nephromopsispseudocomplicata*	China: Yunnan	2017-Aug-20	L60353 (KUN)	OP901598
*Parmotremaperlatum*	Spain: Asturias	-	LF00024 (AMNH)	OP901599
*Parmotremapseudotinctorum*	Spain: Lanzarote	2013	LF00020 (AMNH)	OP901600
*Protousneamagellanica*	Chile: Araucania	2017-Dec-3	0402940 (DUKE)	OP901601
*Tuckermannopsischlorophylla*	Iceland: IAu	1996-Jul-12	LA18869 (AMNH)	OP901602
*Usneaflammea* ^a^	Portugal: Alentejo	2015	LF00029 (AMNH)	OP901603
*Usnealongissimi* ^a^	Russia: Khabarovsk Krai	2013-Jul-30	0339139 (DUKE)	OP901605
*Usneapangiana* ^a^	Japan: Kyushu	2014-Nov-12	0346943 (DUKE)	OP901606
*Usneacavernosa* ^a^	USA: Michigan	2013-Jun-28	0338717 (DUKE)	OP901607
*Usneahimalayana* ^a^	Taiwan: Taichung	2009-Oct-4	0311007 (DUKE)	OP901608
*Usneatrichodeoides* ^a^	Russia: Khabarovsk Krai	2013-Jul-30	0339133 (DUKE)	OP901609
*Usnocetrariaoakesiana*	Norway: Buskerud	2016-Jun-21	L222316 (O)	OP901610
*Usnocetrariaoakesiana*	Norway: Buskerud	2016-Jun-21	L222312 (O)	OP901611
*Vulpicidacanadensis*	USA: California	2013-Jul-28	0332704 (DUKE)	OP901612
*Vulpicidajuniperinus*	Norway: Hedmark	2019-Jul-11	L19277 (O)	OP901613
*Vulpicidajuniperinus*	Norway: Hordaland	2019-Jul-27	L19175 (O)	OP901614
*Vulpicidajuniperinus*	Norway: Sogn og Fjordane	2019-Apr-27	L19052 (O)	OP901615
*Vulpicidapinastri*	Canada: Ontario	2015-Jul-13	015998 (DUKE)	OP901616
*Vulpicidapinastri*	Norway: Sor-Trondelag	2019-Aug-24	L19217 (O)	OP901617
*Vulpicidapinastri*	Norway: Nordland	2019-Aug-6	L19202 (O)	OP901618
*Xanthoparmeliacamtschadalis*	Spain: Castellon	2007	LF00025 (AMNH)	OP901619
*Xanthoparmeliaprotomatrae*	Spain: Castellon	2011	LF00027 (AMNH)	OP901620
*Xanthoparmeliasubdiffluens*	Spain: Castellon	2007	LF00026 (AMNH)	OP901621
*Xanthoparmeliatinctina*	Spain: Castellon	2011	LF00028 (AMNH)	OP901622

^a^: *Usnea* specimens were amplified with alternative primers.

## Supplementary materials

Supplementary material 1 Multiple sequence alignment for fungal mtSSU primer design in the family Parmeliaceae (except for *Usnea*) Data type alignment https://binary.pensoft.net/file/827793This dataset is made available under the Open Database License (http://opendatacommons.org/licenses/odbl/1.0/). The Open Database License (ODbL) is a license agreement intended to allow users to freely share, modify, and use this Dataset while maintaining this same freedom for others, provided that the original source and author(s) are credited.Maonian Xu, Yingkui Liu, Erik Möller, Scott LaGreca, Patricia Moya, Xinyu Wang, Einar Timdal, Hugo de Boer, Eva Barreno, Lisong Wang, Holger Thüs, Ólafur Andrésson, Kristinn Pétur Magnússon, Elín Soffia Ólafsdóttir, Starri Heiðmarsson

Supplementary material 2 Priming sites for alternative mtSSU primers, Nanodrop results and *in silico* PCR amplicons Data type figures, table (word document) https://binary.pensoft.net/file/827794This dataset is made available under the Open Database License (http://opendatacommons.org/licenses/odbl/1.0/). The Open Database License (ODbL) is a license agreement intended to allow users to freely share, modify, and use this Dataset while maintaining this same freedom for others, provided that the original source and author(s) are credited.Maonian Xu, Yingkui Liu, Erik Möller, Scott LaGreca, Patricia Moya, Xinyu Wang, Einar Timdal, Hugo de Boer, Eva Barreno, Lisong Wang, Holger Thüs, Ólafur Andrésson, Kristinn Pétur Magnússon, Elín Soffia Ólafsdóttir, Starri Heiðmarsson

Supplementary material 3 Multiple sequence alignment for fungal mtSSU primer design of the genus *Usnea* Data type alignment https://binary.pensoft.net/file/827795This dataset is made available under the Open Database License (http://opendatacommons.org/licenses/odbl/1.0/). The Open Database License (ODbL) is a license agreement intended to allow users to freely share, modify, and use this Dataset while maintaining this same freedom for others, provided that the original source and author(s) are credited.Maonian Xu, Yingkui Liu, Erik Möller, Scott LaGreca, Patricia Moya, Xinyu Wang, Einar Timdal, Hugo de Boer, Eva Barreno, Lisong Wang, Holger Thüs, Ólafur Andrésson, Kristinn Pétur Magnússon, Elín Soffia Ólafsdóttir, Starri Heiðmarsson
