# Supplementary material for: ﻿Mycobiont-specific primers facilitate the amplification of mitochondrial small subunit ribosomal DNA: a focus on the lichenized fungal genus Melanelia (Ascomycota, Parmeliaceae) in Iceland
Source: MycoKeys. 2023 Mar 21;96:57–75. doi: 10.3897/mycokeys.96.100037 (PMC10210050; doi:10.3897/mycokeys.96.100037)
Supplement: Supplementary material 2 — Priming sites for alternative mtSSU primers, Nanodrop results and in silico PCR amplicons [file mycokeys-96-057-s002.docx]

**Supplementary File 2**


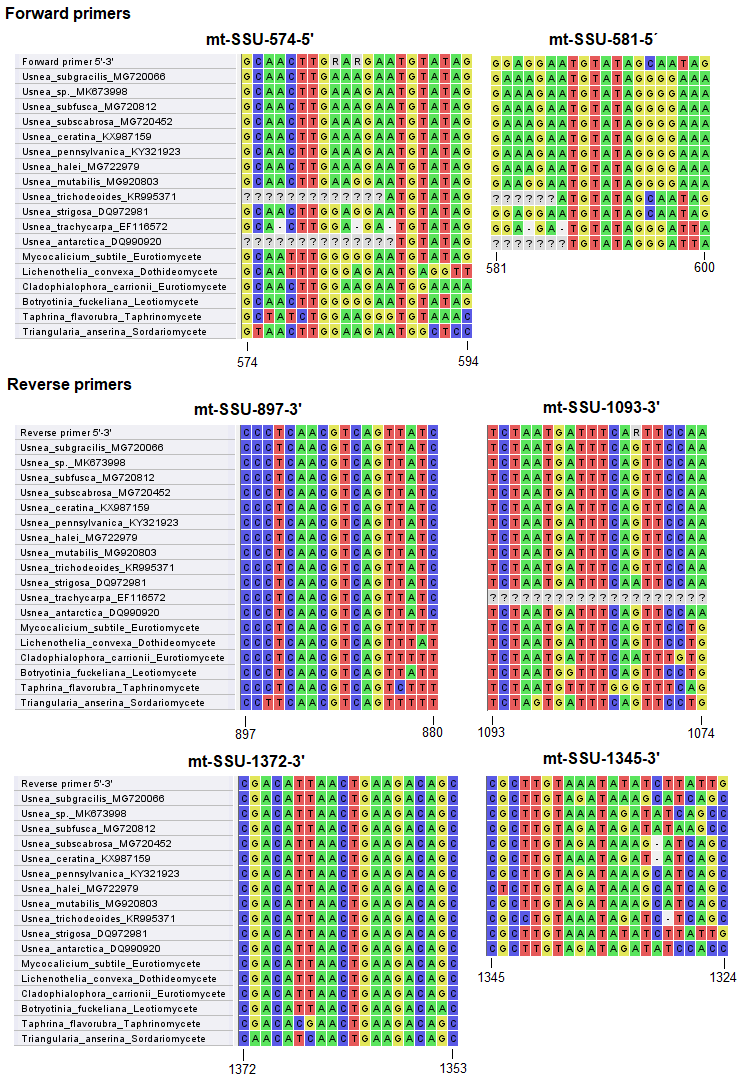


**Fig. S1.** Sequence alignments at the priming sites for alternative primers. From the alignments, we show that our newly designed primer pair, mt-SSU-581-5’and mt-SSU-1345-3’, cannot reliably prime to the *Usnea* mycobiont sequences, especially at the 3’end, and we have to design alternatives primers to cope with PCR amplification. Supplementary File 3 should be consulted while selecting the reverse primers.


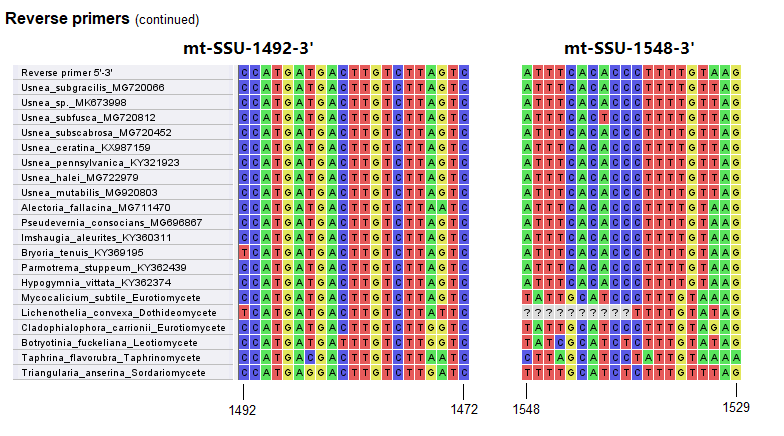

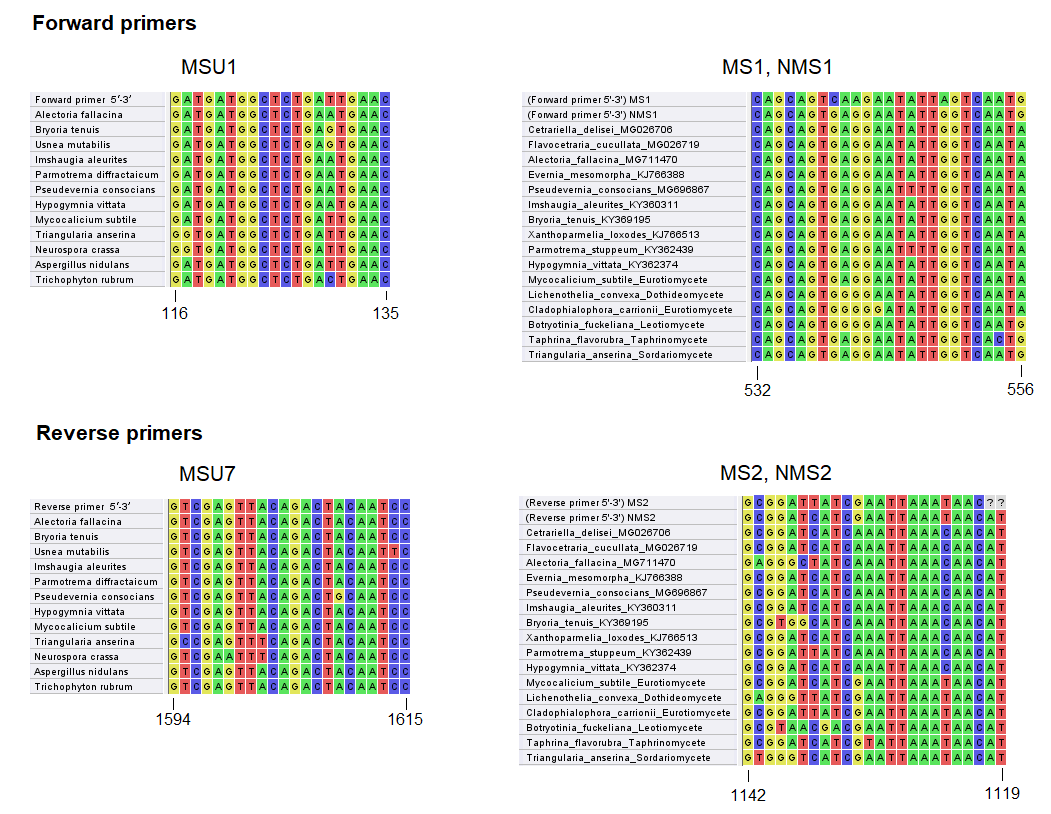


**Fig. S2.** Other published mtSSU primers and their alignments at primer binding sites.


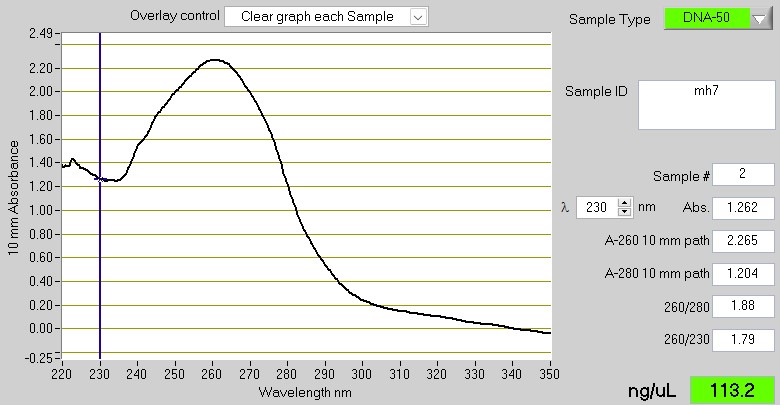


**Fig. S3.** Nanodrop results of the DNA extract from one *Melanelia* specimen which failed in obtaining PCR products. The low value of 260/230 = 1.79 indicates presence of contaminants which absorb at 230 nm. The value of 260/230 should be around 2.0 to 2.2 to be regarded as “pure”.

**Table S1.** Number of *in silico* PCR amplicons in each fungal family using the primer pair mrSSU1&mrSSU3R.

| Family | Number | Family | Number | Family | Number |
| --- | --- | --- | --- | --- | --- |
| Nectriaceae | 589 | Hericiaceae | 5 | Ceratocystidaceae | 1 |
| Aspergillaceae | 201 | Hymenochaetaceae | 5 | Cerrenaceae | 1 |
| Trichocomaceae | 156 | Pertusariaceae | 5 | Chrysotrichaceae | 1 |
| Graphidaceae | 79 | Pneumocystidaceae | 5 | Coccocarpiaceae | 1 |
| Ophiocordycipitaceae | 69 | Pyriculariaceae | 5 | Coniothyriaceae | 1 |
| Ophiostomataceae | 63 | Diaporthaceae | 4 | Corynesporascaceae | 1 |
| Cordycipitaceae | 59 | Hypoxylaceae | 4 | Cryphonectriaceae | 1 |
| Lecanoraceae | 48 | Ploettnerulaceae | 4 | Dothioraceae | 1 |
| Hypocreaceae | 33 | Podosporaceae | 4 | Drepanopezizaceae | 1 |
| **Parmeliaceae** | **33** | Vuilleminiaceae | 4 | Gnomoniaceae | 1 |
| Polyporaceae | 29 | Agaricaceae | 3 | Gomphidiaceae | 1 |
| Teloschistaceae | 27 | Boletaceae | 3 | Herpotrichiellaceae | 1 |
| Cladoniaceae | 20 | Chaetomiaceae | 3 | Hygrophoraceae | 1 |
| Clavicipitaceae | 19 | Cladosporiaceae | 3 | Hyphodiscaceae | 1 |
| Glomerellaceae | 19 | Collemataceae | 3 | Massalongiaceae | 1 |
| Erysiphaceae | 17 | Didymellaceae | 3 | Melanommataceae | 1 |
| Lyophyllaceae | 17 | Icmadophilaceae | 3 | Microascaceae | 1 |
| Omphalotaceae | 13 | Lichenotheliaceae | 3 | Mollisiaceae | 1 |
| Botryosphaeriaceae | 12 | Pannariaceae | 3 | Monoblastiaceae | 1 |
| Marasmiaceae | 11 | Phlyctidaceae | 3 | Morchellaceae | 1 |
| Sclerotiniaceae | 10 | Pluteaceae | 3 | Mycocaliciaceae | 1 |
| Tremellaceae | 10 | Taphrinaceae | 3 | Nephromataceae | 1 |
| unknown | 10 | Tricholomataceae | 3 | Peltulaceae | 1 |
| Arthrodermataceae | 9 | Tuberaceae | 3 | Peniophoraceae | 1 |
| Lobariaceae | 9 | Bulleribasidiaceae | 2 | Phaeosphaeriaceae | 1 |
| Orbiliaceae | 9 | Helotiaceae | 2 | Placynthiaceae | 1 |
| Umbilicariaceae | 9 | Leptosphaeriaceae | 2 | Psoraceae | 1 |
| Acarosporaceae | 8 | Opegraphaceae | 2 | Pyrenulaceae | 1 |
| Sordariaceae | 8 | Pisolithaceae | 2 | Pyronemataceae | 1 |
| Mycosphaerellaceae | 7 | Pseudeurotiaceae | 2 | Saccotheciaceae | 1 |
| Peltigeraceae | 7 | Rhizopogonaceae | 2 | Sarocladiaceae | 1 |
| Physciaceae | 7 | Roccellaceae | 2 | Schizoporaceae | 1 |
| Plectosphaerellaceae | 7 | Sphaerophoraceae | 2 | Shiraiaceae | 1 |
| Pleosporaceae | 7 | Stereocaulaceae | 2 | Sporocadaceae | 1 |
| Ramalinaceae | 7 | Trypetheliaceae | 2 | Stictidaceae | 1 |
| Russulaceae | 7 | Verrucariaceae | 2 | Suillaceae | 1 |
| Trichosporonaceae | 7 | Apiosporaceae | 1 | Thelebolaceae | 1 |
| Arthoniaceae | 6 | Astrosphaeriellaceae | 1 | Triblidiaceae | 1 |
| Ajellomycetaceae | 5 | Auriculariaceae | 1 | Trichomeriaceae | 1 |
| Amanitaceae | 5 | Boletinellaceae | 1 | Xylariaceae | 1 |
| Bionectriaceae | 5 | Bondarzewiaceae | 1 |  |  |
